# Loss of ACOX1 in clear cell renal cell carcinoma and its correlation with clinical features

**DOI:** 10.1515/biol-2022-0696

**Published:** 2023-09-08

**Authors:** Yingxi Mo, Jun Zhao, Ran Zhao, Yiying Huang, Ziyuan Liang, Xiaoying Zhou, Jiemei Chu, Xinli Pan, Siyu Duan, Shiman Chen, Liufang Mo, Bizhou Huang, Zhaozhang Huang, Jiale Wei, Qian Zheng, Wenqi Luo

**Affiliations:** Department of Research, Guangxi Medical University Cancer Hospital, Nanning, China; Key Laboratory of High-Incidence-Tumor Prevention & Treatment, Guangxi Medical University, Ministry of Education, Nanning, China; Affiliated Stomatological Hospital of Guangxi Medical University, Nanning, China; Life Science Institute, Guangxi Medical University, #22 Shuangyong Road, Nanning, 530021, China; Guangxi Key Laboratory of Marine Natural Products and Combinatorial Biosynthesis Chemistry, Guangxi Academy of Sciences, Nanning, China; Department of Pathology, Guangxi Medical University Cancer Hospital, 530021, Nanning, China

**Keywords:** clear cell renal cell carcinoma, ACOX1, miR-16-5P, bioinformatics

## Abstract

Clear cell renal cell carcinoma (ccRCC) is a major pathological type of kidney cancer with a poor prognosis due to a lack of biomarkers for early diagnosis and prognosis prediction of ccRCC. In this study, we investigated the aberrant expression of Acyl-coenzyme A oxidase 1 (ACOX1) in ccRCC and evaluated its potential in diagnosis and prognosis. ACOX1 is the first rate-limiting enzyme in the peroxidation β-oxidation pathway and is involved in the regulation of fatty acid oxidative catabolism. The mRNA and protein levels of ACOX1 were significantly downregulated in ccRCC, and its downregulation was closely associated with the tumor-node-metastasis stage of patients. The ROC curves showed that ACOX1 possesses a high diagnostic value for ccRCC. The OS analysis suggested that lower expression of ACOX1 was closely related to the worse outcome of patients. In addition, gene set enrichment analysis suggested that expression of ACOX1 was positively correlated with CDH1, CDH2, CDKL2, and EPCAM, while negatively correlated with MMP9 and VIM, which strongly indicated that ACOX1 may inhibit the invasion and migration of ccRCC by reversing epithelial-mesenchymal transition. Furthermore, we screened out that miR-16-5p is upregulated at the mRNA transcript level in ccRCC and negatively correlated with ACOX1. In conclusion, our results showed that ACOX1 is abnormally low expressed in ccRCC, suggesting that it could serve as a diagnostic and prognostic biomarker for ccRCC. Overexpression of miR-16-5p may be responsible for the inactivation of ACOX1.

## 1 Introduction

Approximately 430,000 cases of renal cell carcinoma (RCC) are diagnosed each year, with clear cell renal cell carcinoma (ccRCC) being the main pathological type of RCC. [1]. Despite recent advances in diagnostic methods, the incidence of RCC increases every year, and a significant proportion of patients are diagnosed at an advanced stage, with 17% of patients having distant metastases [2]. Although surgical resection improves the 5-year survival rate in ccRCC, the overall prognosis remains poor [3,4]. To date, although surgery remains the gold standard of treatment for ccRCC, approximately one-third of patients will still have a recurrence after surgery. Therefore, there is an urgent need to find new ways to treat the disease [5,6]. As research into genes and mRNAs deepens, microRNAs help contribute to improve the understanding of cancer development and targeted therapies [7,8]. Therefore, the identification of novel biomarkers for diagnosis and personalized treatment of ccRCC is urgently needed.

A common feature of cancer cells is the ability to rewire their metabolism to sustain the production of ATP and macromolecules needed for cell survival and proliferation [9]. In recent years, lipid metabolism in tumorigenesis has been extensively studied. As lipids provide essential materials for cellular membrane formation and realize other functions for proliferating tumor growth, such as phosphatidylcholine (PC) and phosphatidylethanolamine (PE). Cancer cells use de novo synthesis of fatty acids (FAs) for signal transduction, building the cellular membrane, or energy supply [10,11]. For example, FAs stored within lipid droplets provide adenosine triphosphate (ATP) molecules through β-oxidation, mainly serving as an energy source for cell proliferation [9,12]. β-Oxidation takes place in the mitochondria and peroxisomes and is generally considered to be part of cancer metabolism [13]. In addition to ATP synthesis, β-oxidation is also involved in synthesizing NAPDH in the cytoplasm, thereby facilitating the de novo synthesis of fatty acids by cancer cells. Numerous studies have shown that abnormalities in β-oxidation are closely linked to tumorigenesis and cancer cells rely on their reprogrammed metabolism for proliferation, survival, and metastasis [14].

Acyl-coenzyme A oxidase 1 (ACOX1), the first rate-limiting enzyme for fatty acid β-oxidation (FAO), is a highly conserved enzyme and is mainly expressed in the liver, followed by the kidney, brain, and adipose tissue [15]. FAO takes place in mitochondria and peroxisomes [13], and its abnormalities are associated with tumorigenesis [14]. Peroxisomes are ubiquitous organelles in eukaryotic cells involved in several metabolic pathways, such as FAO, bile acid synthesis, and purine degradation, etc. [16]. As performing the first step of peroxisomal β-oxidation, which is the major enzymatic step [17], an aberrant expression of ACOX1 has been implicated in a variety of cancers. It has been demonstrated that the upregulation of ACOX1 in lymphoma promotes cancer cell proliferation, while its downregulation inhibits proliferation and induces apoptosis [18]. Knocking down ACOX1 promotes lipid hydrolysis by inducing autophagy in the liver, thereby preventing hepatic steatosis. ACOX1 might serve as a new therapeutic option for treating nonalcoholic fatty liver disease (NAFLD) [19].

Herein, we aimed to demonstrate the differential expression of ACOX1 in ccRCC and evaluate its diagnostic and prognostic value in ccRCC. In addition, we tried to reveal the molecular mechanism for the aberrant expression of ACOX1 in ccRCC, from the aspect of miRNAs.

## 2 Materials and methods

### 2.1 Cell lines and tissue samples

The human renal epithelial cell line 293T and ccRCC cell lines 786-O and Caki-2 were procured from Procell Life Science & Technology Co., Ltd. (Wuhan, China). The 293T human renal epithelial cells were cultured in Dulbecco’s Modified Eagle Medium (DMEM; Gibco, USA) supplemented with 10% fetal bovine serum (FBS; Invitrogen) and penicillin (100 U/ml). The human ccRCC cell lines 786-O (CL-0010, China) were maintained in 1640 medium (Invitrogen, Carlsbad, CA, USA) supplemented with 10% FBS and penicillin (100 U/ml). The Caki-2 (CL-0326, CHINA) was cultured in McCoy’s 5a Medium Modified supplemented with 10% FBS and penicillin (100 U/ml). All cell lines used in this study were cultured under standard culture conditions (5% CO_2_, 37°C).

The complementary deoxyribonucleic acid (cDNA) tissue array (Cat no: me cDNA-HKidE030CS01, Shanghai OUTDO Biotech Co., Ltd.) used in this study included 15 ccRCC samples and 15 matched precancerous tissue samples. All 15 patients were enrolled in this study based on pathological examination, and all patients provided written informed consent.

Sixty-four formalin-fixed, paraffin-embedded ccRCC tissues and para-carcinoma tissues were obtained from patients at Guangxi Medical University Cancer Hospital (Nanning, Guangxi, China). Complete clinicopathological and follow-up data are available for 15 ccRCC patients (nine males and six females).


**Informed consent:** Informed consent has been obtained from all individuals included in this study.
**Ethical approval:** The research related to human use has been complied with all the relevant national regulations, institutional policies, and in accordance with the tenets of the Helsinki Declaration, and has been approved by the Ethics Committees of Guangxi Medical University Cancer Hospital (Nanning, China [LW2020043]).

### 2.2 TCGA database

The mRNA-seq expression data were downloaded from the University of California, Santa Cruz Xena browser (https://xenabrowser.net/), including 534 ccRCC tissues and 72 normal tissue samples. On the other hand, corresponding clinicopathological parameters include age, sex, tumor-node-metastasis stage, pathological stage, and tumor diameter.

### 2.3 Gene expression omnibus (GEO) database

We searched ccRCC-relevant microarray data sets of ACOX1 from the National Center of Biotechnology Information Gene Expression Omnibus (https://www.ncbi.nlm.nih.gov/) to examine the role of ACOX1 in ccRCC. By using the following terms: (renal OR kidney) AND (cancer OR carcinoma* OR tumor OR tumor OR tumors OR tumors OR neoplasm* OR malignance* OR oncology* OR KIRC OR ccRCC OR RCC). The inclusion criteria were as follows: (1) human tissue samples, (2) no less than four cancer and non-cancer samples, and (3) data sets containing ACOX1 mRNA expression data. The meta-analysis was performed using StataSE-64 software.

### 2.4 The University of ALabama CANcer (UALCAN) database

UALCAN (http://ualcan.path.uab.edu/) is a public online available database for analyzing the cancer omics data (TCGA, MET500, Clinical Proteomic Tumour Analysis Consortium (CPTAC), and Children’s Brain Tumour Tissue Consortium (CBTTC). We used the database to explore transcription and expression levels of ACOX1 in ccRCC and normal tissues.

### 2.5 RNA isolation and Quantitative Real-time reverse transcription polymerase chain reaction (qRT-PCR)

Total RNA was prepared with TRIZol reagent (Invitrogen, Carlsbad, CA, USA) and reverse transcribed into cDNA with PrimeScript RT-PCR kit (Takara Bio, code No. 15596-026, USA). RR036A, Japan). Quantitative RT-PCR was then performed on a QuantStudio 5 Flex Real-Time PCR System (Thermo Fisher Scientific, Waltham, MA, USA) using the SYBR Green quantitative PCR kit (#A25742, Thermo Fisher Scientific, Waltham, MA, USA). The Ct (2^−ΔΔCt^) method was used to calculate the ACOX1 mRNA transcript level. The reference gene for quantification assays was β-actin. The sequences of the qRT-PCR primers are as follows:

- ACOX1 Forward: 5′-CCCATAAGCCTTTGCCAGGA-3′;
- ACOX1 Reverse: 5′-GGCTTCACCTGGGCATACTT-3′;
- β-actin-Forward: 5′-GCTCAGACACCATGGGGAAG-3′;
- β-actin-Reverse: 5′-TGTAGTTGAGGTCAATGAAGGGG-3′.

### 2.6 Immunohistochemistry (IHC) staining

Paraffin-embedded ccRCC tissues were cut into 4-μm thick sections. An SP-9000 detection kit (ZSGB-Bio, Beijing, China) was used to perform the IHC staining. The slides were subsequently baked at 60°C for 1 h, deparaffinized, and rehydrated in xylene and decreasing concentrations of ethanol, followed by antigen retrieval in 10 mM Tris–citrate buffer (pH of 7.0) in a pressure cooker. Endogenous peroxidase activity of the sections was blocked by a streptavidin-peroxidase staining kit (No. SP-9000; Zsbio, China), and each section was then incubated with 5% normal goat serum for 30 min at room temperature to block nonspecific bindings. Subsequently, the slides were incubated with a primary ACOX1 antibody (Cat No. 10957-1-AP, Wuhan Sanying [diluted 1:200]) at 4°C overnight. After washing the primary antibody with phosphate-buffered saline, the slides were then incubated with a secondary antibody (SP-9000, ZSGB-BIO, Beijing, China), at room temperature for 10 min. Finally, the slides were developed with 3,3′-diaminobenzidine (DAB, ZLI9018ZSGB-BIO, Beijing, China), and hematoxylin counterstaining, dehydrating, clearing, and mounting were performed.

### 2.7 Evaluation of IHC staining

IHC staining results were confirmed by at least two experienced pathologists in a double-blind manner. The final IHC scores were the product of the “positive staining rate score” and “staining intensity score.” The positive staining rate score was recorded based on the percentage of positive staining cells as follows: <10% is 0, 10–50% is 1, 51–75% is 2, and ≥75% is 3. The intensity of cell staining was defined as follows: 0, negative; 1, light yellow; 2, brown or yellow; and 3, brown. The percentage and intensity scores of the five random fields were multiplied. Finally, the staining score was graded as 2–3, weakly positive (+); 4–6, moderately positive (++); 7–9, strongly positive (+++); and ≥9 (+), all positive expressions. Images were obtained using an Olympus microscope (Olympus BX53, Japan).

### 2.8 Gene expression profiling interactive analysis (GEPIA)

GEPIA (http://gepia2.cancer-pku.cn/#index) online database contains RNA sequencing data from TCGA and GTEX, including 9,736 tumor tissues and 8,587 normal tissue samples. We used GEPIA to generate a overall survival curve of ACOX1 to explore the effect of ACOX1 expression on the prognosis of patients with ccRCC.

### 2.9 Gene set enrichment analysis (GSEA)

GSEA was performed on the dataset (534 ccRCC samples and 72 matched precancerous samples) from TCGA. The data set (h.all. v6.1.symbols) was categorized into high- and low-expression groups based on the median ACOX1 gene expression, and the signaling mechanism involved was analyzed in the ACOX1 overexpression group by using GSEA 4.2.3. Statistical significance was considered when the false discovery rate (FDR) was <0.25, and the *P*-value of the normalized enrichment score (NES) was <0.05.

### 2.10 Prediction of miRNAs potentially targeting the ACOX1mRNA

Targeted prediction of potential miRNAs of ACOX1 was performed using four public databases: miRDB (http://mirdb.org/), Tarbase (https://dianalab.e-ce.uth.gr/html/diana/web/index.php? *r* = tarbasev8%2Findex), ENCORI (https://starbase.sysu.edu.cn/), and TargetScan (https://www.targetscan.org/vert_72/).

### 2.11 Statistical analysis

In this study, IBM SPSS 23.0 and GraphPad Prism 8.0 were used for data analysis. The data were presented as the mean ± standard deviation (*x* ± *s*) in datasets. A *t*-test was used to analyze the expression of ACOX1 in the ccRCC and paracancerous samples from the TCGA and GEO databases. The meta-analysis was performed using Stata-SE-64 12.0 statistical software and the diagnostic value of the ACOX1 gene expression in ccRCC was evaluated based on the ROC curve. Survival analysis was performed using GEPIA. Kaplan–Meier survival analysis and univariate and multivariate Cox regression analyses were used to analyze the relationship between ACOX1 expression and poor prognosis in patients with ccRCC. Statistical significance was set at *P* < 0.05.

## 3 Results

### 3.1 Expression of ACOX1 significantly downregulated in ccRCC

**Figure 1:**
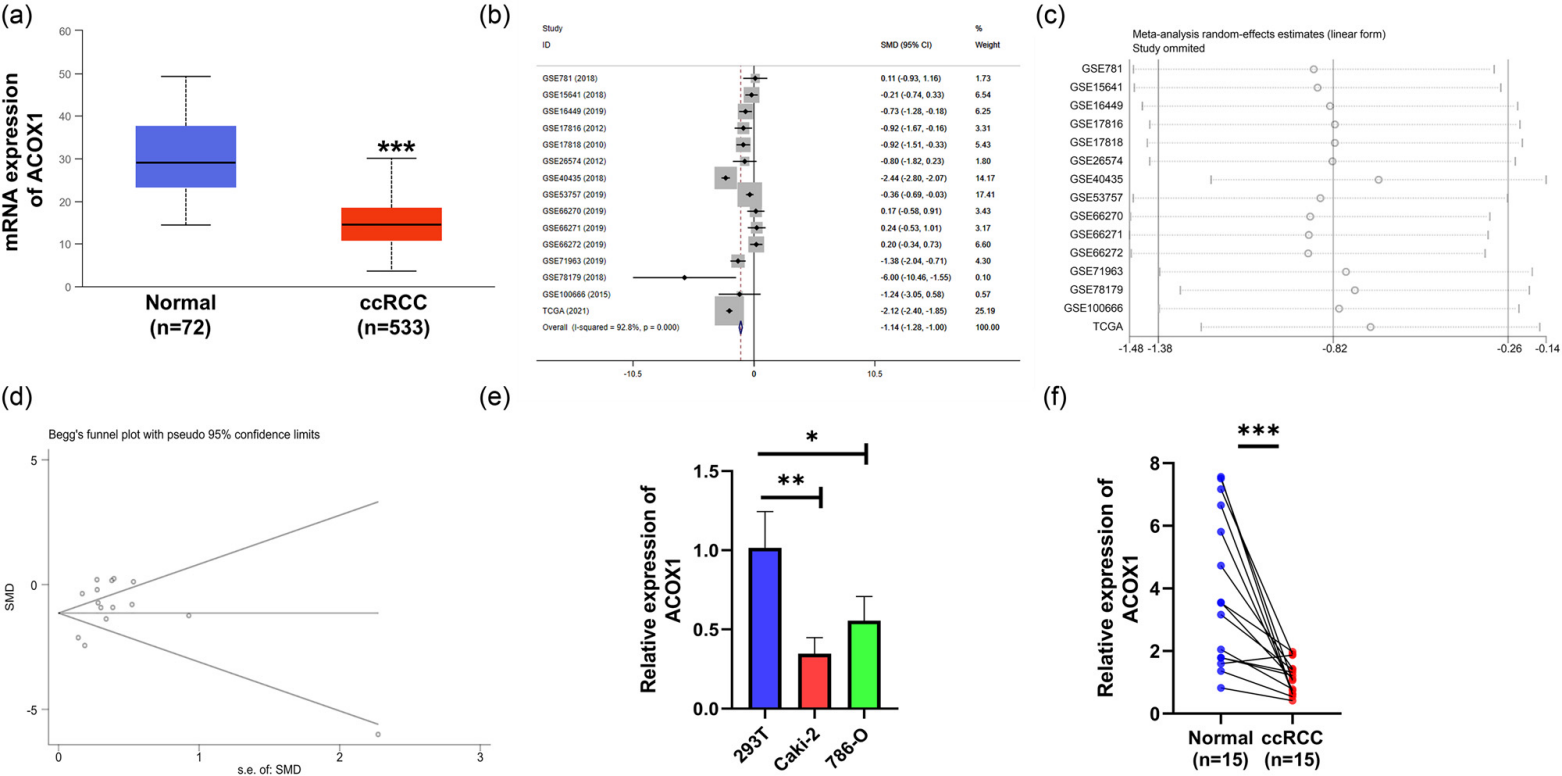
The transcriptional level of ACOX1 in ccRCC. (a) ACOX1 mRNA expression in 533 ccRCC tissues and 72 normal tissues in the TCGA database. (b) Forest plot of mRNA transcript levels of ACOX1 in ccRCC. (c) Sensitivity analysis of the ACOX1 in ccRCC. (d) Begg funnel plot analysis of ACOX1 expression in ccRCC. (e) ACOX1 transcript levels in ccRCC cell lines and immortalized cell 293 T. (f) mRNA transcript levels in 15 ccRCC primary tumor tissues and matched paraneoplastic tissues (*P < 0.05, **P < 0.01, ***P < 0.001).

To discover the changes in ACOX1 in ccRCC, based on the TCGA database, a significant down-regulation of ACOX1 was observed in patients with ccRCC (*n* = 533) compared with the normal control tissues (*n* = 72 [10.52 ± 0.41 vs 11.43 ± 0.63, *P* < 0.01]; Figure 1a).

Next, we performed a meta-analysis of the microarrays containing ACOX1 data in the GEO database. The analysis revealed that ACOX1 transcriptional levels were downregulated in 507 ccRCC tissues and 325 normal tissues (*P* = 0.000, I2 = 92.8%). There was significant heterogeneity (Figure 1b), although sensitive sexuality analysis verified that the expression of ACOX1 was downregulated in ccRCC (95% CI: −1.38 to −0.26, *P* = 0.000 [Figure 1c]). Simultaneously, Beggar’s funnel plot was used to evaluate publication bias, and the results revealed no publication bias (*P* = 0.428 > 0.05 [Figure 1d]).

**Figure 2:**
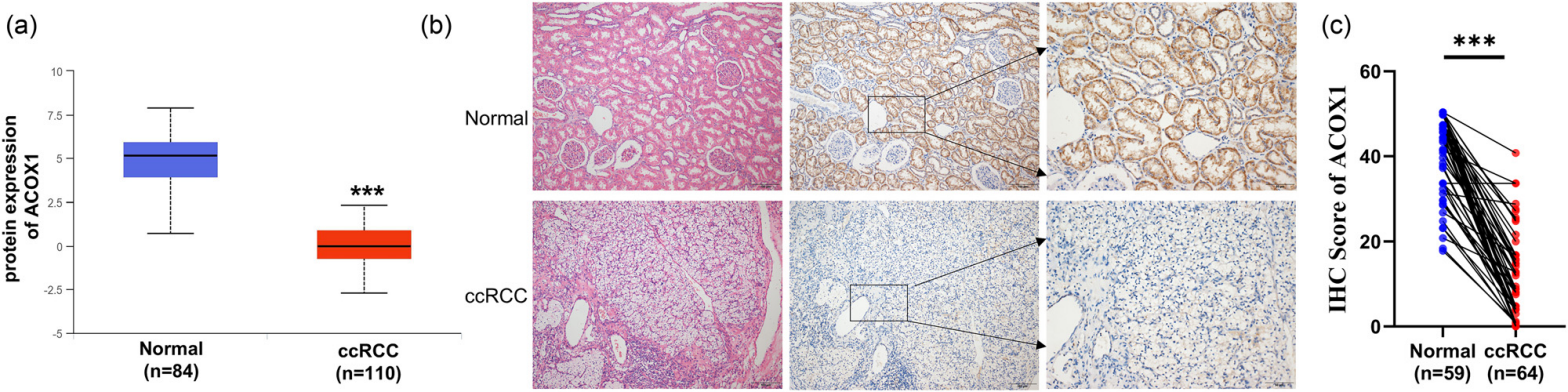
The protein expression level of ACOX1 in ccRCC. (a) Protein expression levels of ACOX1 in 110 ccRCC tissues and 84 normal tissues. (b) Representative HE staining and IHC staining of ACOX1 in ccRCC and normal tissues. (c) Statistical analysis data of IHC staining score.

Further, to evaluate whether its transcript expression levels were altered, we verified the transcript levels of ACOX1 in ccRCC cell lines and tissues by real-time PCR. A downregulation of mRNA transcription was found in Caki-2 and 786-O renal cancer cell lines compared to normal kidney 293T cell lines (Figure 1e). Also, downregulation of ACOX1 was found in tissue microarrays of 15 paired ccRCCs (Figure 1f).

In addition, using the CPTAC database, we observed that ACOX1 protein expression levels were significantly reduced in ccRCC tissues (*n* = 110) compared to normal tissues (*n* = 84; Figure 2a). IHC was used to evaluate ACOX1 protein levels in ccRCC tissues (*n* = 59) and normal tissues (*n* = 64; Figure 2b). Our results revealed that ACOX1 expression is significantly higher in normal tissues than in ccRCC tissues (Figure 2c). A lower ACOX1-positive rate was observed in 35 of 64 ccRCC tissues, while positive expression of ACOX1 was 47.5% (28 of 59) in normal tissue, suggesting ACOX1 downregulation in ccRCC (*χ* ^2^ = 0.057, *P* = 0.858). ccRCC patients with N (*n* = 4) and M stages (*n* = 6) exhibited prominently lower levels of ACOX1 expression (*P* < 0.05; Table 2).

### 3.2 ACOX1 is an effective biomaker for the diagnosis and prognosis of ccRCC patients

**Figure 3:**
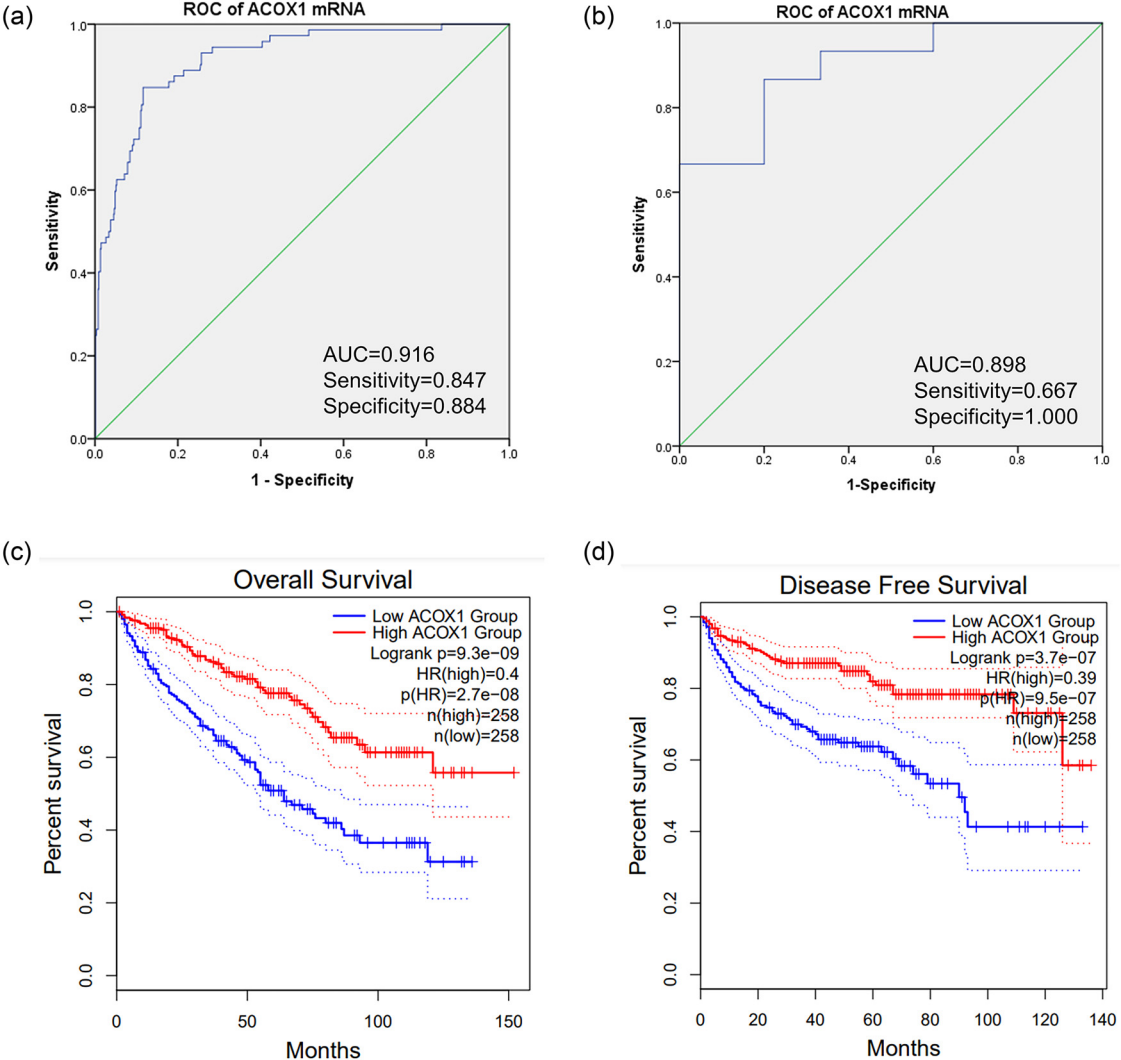
ACOX1 mRNA expression in ccRCC and correlated with diagnosis, and prognosis. (a) ROC curve of ACOX1 expression in 533 ccRCC tissues and 72 normal tissues based on TCGA database. (b) ROC curve of ACOX1 expression in 15 ccRCC tissues and matched paraneoplastic tissues from cDNA microarrays. (c) Analysis of the relationship between the mRNA expression level of ACOX1 and overall survival based on TCGA data. (d) Analysis of the relationship between the mRNA expression level of ACOX1 and disease-free survival of ccRCC patients based on TCGA data.

The ROC curve was used to assess the diagnostic value of ACOX1 in ccRCC. We found that the area under the curve (AUC) was 0.916 (*P* < 0.001) based on the TCGA database (Figure 3a). In ccRCC cDNA, its AUC was 0.898 (*P* > 0.001; Figure 3b). These findings suggested that ACOX1 could serve as a clinical diagnostic molecular marker for ccRCC. Figure 3c and d shows that the overall survival and disease-free survival analysis (DFS) of ACOX1 expression were performed using the GEPIA database. The plots indicated that ccRCC patients with a decreased ACOX1 expression had poor OS (log-ranch test, *P* = 3.4 × 10^−7^; Figure 3c). The OS rate (log-ranch test, *P* = 0.0007) and disease-free survival (DFS: HR = 9.5 × 10^−7^) are shown in Figure 3d. Thus, our data suggest that ACOX1 could serve as a prognostic molecular marker for ccRCC.

Based on clinical data from ccRCC patients in TCGA, we found that ACOX1 expression was lower in male patients (*n* = 345) than in female patients (*n* = 188) (10.49 ± 0.41 vs. 10.57 ± 0.41, *P* < 0.05). In the stage of patients, mRNA levels of ACOX1 were expressed at a lower level in patients with ccRCC in T3–T4 (*n* = 191) than in patients with T1–T2 (*n* = 342 [10.44 ± 0.38 vs. 10.57 ± 0.43]). Meanwhile, in terms of distant metastases and pathological staging, patients with stage M (*n* = 79) were lower than those with the non-M stage (*n* = 422 [10.38 ± 0.39 vs. 10.56 ± 0.41, *P* < 0.05]). And patients with stages III–IV (*n* = 207) were also lower than those with stages I–II (*n* = 324 [10.43 ± 0.38 vs. 10.58 ± 0.42, *P* < 0.05]). However, no significant differences in ACOX1 expression levels were observed between other clinicopathological features such as age and lymphatic metastases (Table 1). Analysis of the clinical information of the patients in the IHC revealed that ACOX1 was only significantly altered in distant metastases, as shown in Table 2 (*P* = 0.046).

**Table 1:** Correlation of ACOX1 mRNA expression with clinicopathological parameters of patients with ccRCC based on the TCGA database

		Relevant expression of ACOX1			
Clinicopathological parameters		*n*	Mean ± SD	*t*	*P*-value
Tissue	Normal	72	11.43 ± 0.63	−13.955	<0.001*
	ccRCC	533	10.52 ± 0.41		
Age	<60	245	10.53 ± 0.40	0.605	0.545
	≥60	288	10.51 ± 0.43		
Gender	Male	345	10.49 ± 0.41	−2.203	0.028*
	Female	188	10.57 ± 0.41		
T	T1–T2	342	10.57 ± 0.43	3.562	<0.001***
	T3–T4	191	10.44 ± 0.38		
N	No	240	10.55 ± 0.42	1.747	0.082
	Yes	16	10.36 ± 0.35		
M	No	422	10.56 ± 0.41	3.608	<0.001***
	Yes	79	10.38 ± 0.39		
Pathologic stage	I–II	324	10.58 ± 0.42	4.082	<0.001***
	III–IV	207	10.43 ± 0.38		

SD, standard deviation; ccRCC, clear cell renal clear cell carcinoma; T, tumor; N, lymph node; M, metastasis.

*P < 0.05 was considered statistically significant.

**Table 2:** Correlation of ACOX1 expression with clinicopathologic characteristics in patients with ccRCC IHC staining

			Relevant expression of ACOX1			
Clinicopathological parameters		Case	Low/*n* (%)	High/*n* (%)	*X* ^2^	*P*-value
Tissue	ccRCC	64	35 (54.7)	29 (45.3)	0.057	0.858
	Adjacent non-cancerous kidney	59	31 (52.5)	28 (47.5)		
Age	<60	42	27 (64.3)	15 (35.7)	0.166	0.683
	≥60	22	13 (59.1)	9 (40.9)		
Gender	Male	38	25 (65.8)	13 (34.2)	0.432	0.511
	Female	26	15 (57.7)	11 (42.3)		
T	T1–T2	42	27 (64.3)	15 (35.7)	0.188	0.665
	T3–T4	22	13 (59.1)	9 (40.9)		
N	No	60	40 (66.7)	20 (33.3)	4.551	0.033
	Yes	4	0 (0.00)	4 (100.0)		
M	No	58	39 (67.2)	19 (32.8)	3.972	0.046*
	Yes	6	1 (16.7)	5 (83.3)		
Pathologic stage	I–II	42	29 (69.0)	13 (31.0)	2.235	0.135
	III–IV	22	11 (50.0)	11 (50.0)		
Tumor diameter	<5 cm	31	20 (64.5)	11 (35.5)	0.336	0.562
	≥5 cm	28	16 (57.1)	12 (42.9)		

SD, standard deviation; ccRCC, clear cell renal clear cell carcinoma; T, tumor; N, lymph node; M, metastasis.

*P < 0.05 was considered statistically significant.

### 3.3 Enrichment analysis of ACOX1 in the ccRCC-related signaling pathways

GSEA was used to evaluate the functional and signaling pathways associated with ACOX1. About 21 criteria for the most enriched signaling pathways that were included. The main transduction pathways involved in ACOX1 associated with ccRCC were as follows: epithelial–mesenchymal transition (EMT), myelocytomatosis (MYC)-targets V1 and MYC targets V2 related to proliferation, TNF-α/NF-kB signaling pathway, the P53 pathway, and the apoptotic pathway (Table 3). At the same time, the correlation between ACOX1 and EMT-related genes was analyzed, and a good correlation was found (Figure 5). It is assumed that ACOX1 expression is associated with the above-mentioned signaling pathways, i.e., activation of this series of signaling pathways in ccRCC may occur through the ACOX1 downregulation.

**Figure 4:**
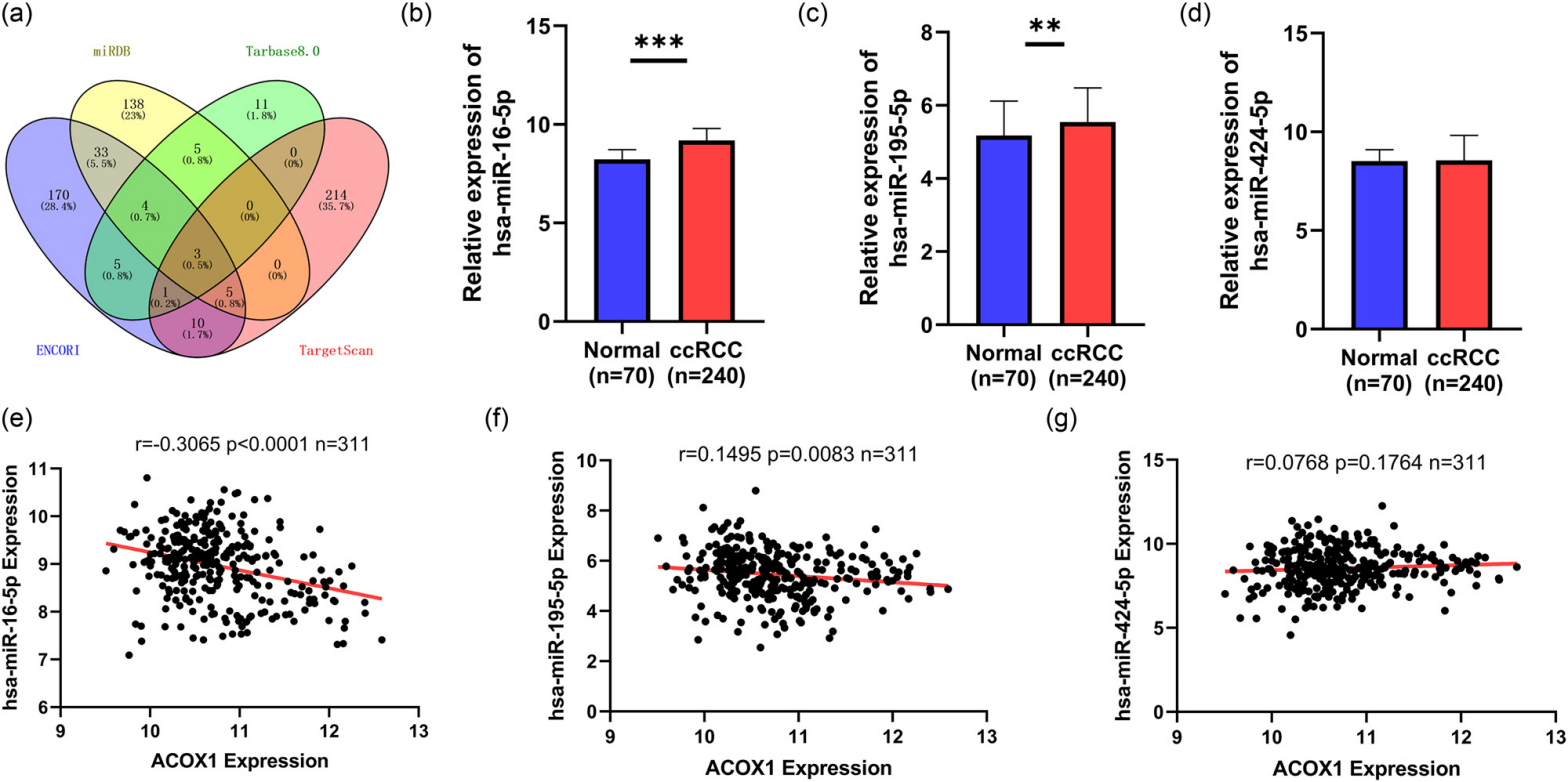
miR-16-5P may target the regulation of ACOX1 in ccRCC. (a) Three microRNAs were predicted to target the regulation of ACOX1 using miRDB,Tarbase8.0, ENCORI, and TargetScan. (b–d) Analysis of mRNA levels of three microRNAs in ccRCC based on the TCGA database in 240 ccRCC tissues and 70 normal tissues. (e–g) Correlation of three microRNAs with ACOX1 in ccRCC.

### 3.4 MiR-16-5p involved in the horizontal expression of ACOX1 mRNA in ccRCC

As shown in Figure 4a, we predicted three miRNAs that might regulate ACOX1 expression based on miRDB (http://mirdb.org/), Tarbase8.0 (https://dianalab.e-ce.uth.gr/html/diana/web/index.php? *r* = tarbasev8%2Findex), ENCORI (https://starbase.sysu.edu.cn/), and TargetScan (https://www.targetscan.org/vert_72/) databases.

**Figure 5:**
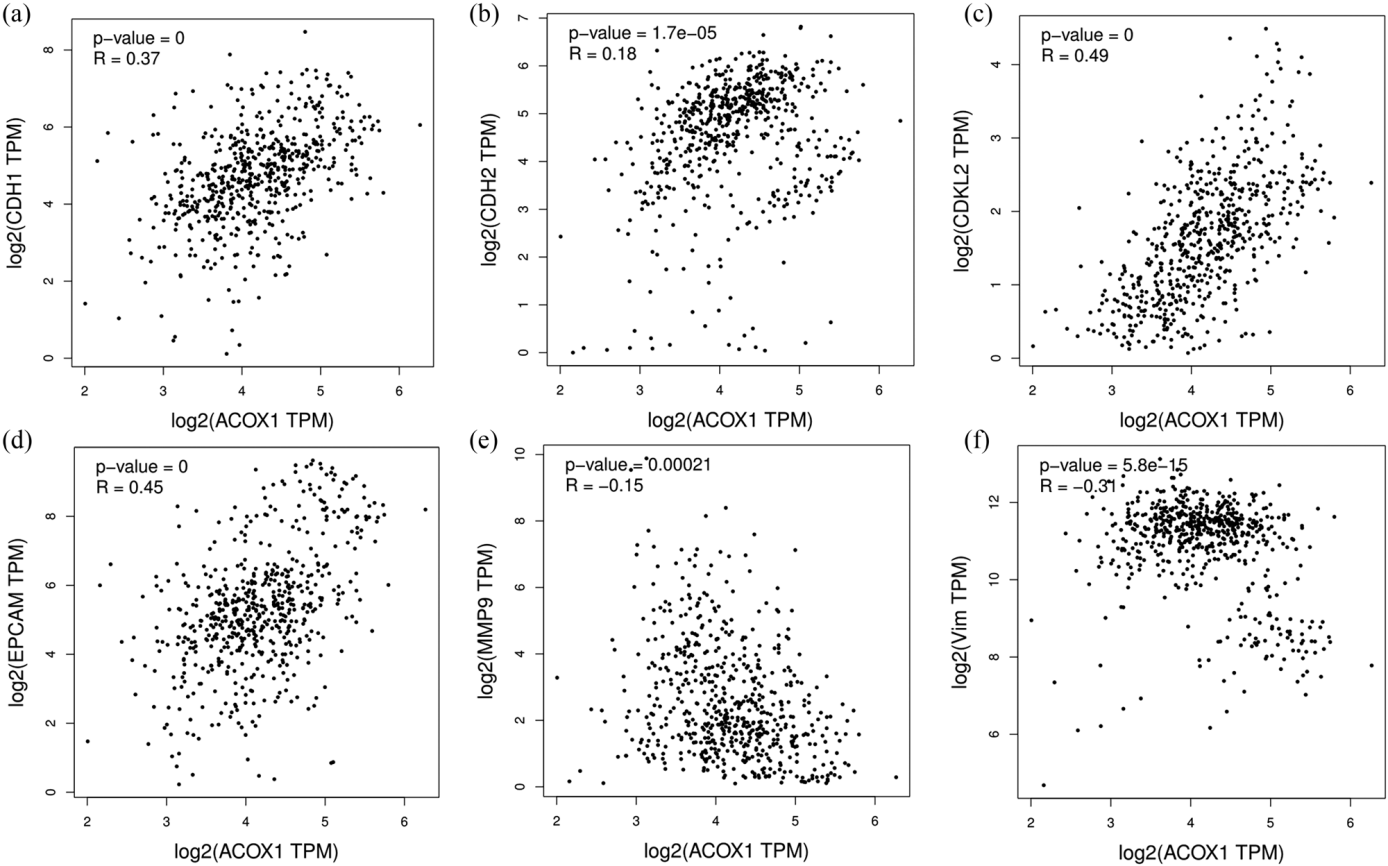
Correlation of ACOX1 mRNA transcript levels in ccRCC with EMT-related genes. (a–f) Based on the TCGA database, the expression of ACOX1 with CDH1, CDH2, CDKL2, EPCAM, MMP9, and VIM was analyzed.

We further analyzed the expression data of the three miRNAs through TCGA and found that all three miRNAs were highly expressed in ccRCC (Figure 4b–d). Only miR-16-5p was negatively correlated with ccRCC (*P* < 0.001), while the rest were positively correlated with ccRCC (Figure 4e–g). It is assumed that miR-16-5p might act as an upstream regulator to suppress the expression of the ACOX1 gene in ccRCC.

In addition, the mRNA transcript levels of miR-16-5p were analyzed for comparison with the clinical information obtained from TCGA. MiR-16-5p expression was significantly lower in ccRCC (*n* = 240) tissues than in normal tissues (*n* = 70, *P* < 0.0001; Table 4). T-stage, lymphatic infiltration, and distant metastasis were not significantly associated with the miR-16-5p expression level.

**Table 3:** Enrichment analysis of signaling pathways involved in the regulation of ACOX1

GS follows link to MSigDB	SIZE	ES	NES	NOM *P*-value	FDR *q*-value
ALLOGRAFT_REJECTION	195	−0.59	−3.01	0.000	0.000
INTERFERON_GAMMA_RESPONSE	196	−0.52	−2.68	0.000	0.000
EPITHELIAL_MESENCHYMAL_TRANSITION	194	−0.51	−2.61	0.000	0.000
E2F_TARGETS	187	−0.49	−2.45	0.000	0.000
INTERFERON_ALPHA_RESPONSE	92	−0.53	−2.41	0.000	0.000
INFLAMMATORY_RESPONSE	197	−0.46	−2.34	0.000	0.000
TNFA_SIGNALING_VIA_NFKB	197	−0.45	−2.29	0.000	0.000
G2M_CHECKPOINT	184	−0.42	−2.16	0.000	0.000
IL6_JAK_STAT3_SIGNALING	87	−0.48	−2.15	0.000	0.000
WNT_BETA_CATENIN_SIGNALING	42	−0.52	−1.99	0.000	0.000
HYPOXIA	190	−0.39	−1.97	0.000	0.000
ANGIOGENESIS	36	−0.51	−1.87	0.002	0.001
APICAL_JUNCTION	193	−0.36	−1.82	0.000	0.001
MYC_TARGETS_V1	188	−0.35	−1.76	0.000	0.001
MYC_TARGETS_V2	58	−0.42	−1.72	0.000	0.002
DNA_REPAIR	139	−0.35	−1.72	0.000	0.002
P53_PATHWAY	190	−0.34	−1.7	0.000	0.002
COMPLEMENT	195	−0.31	−1.6	0.002	0.007
IL2_STAT5_SIGNALING	194	−0.31	−1.59	0.000	0.007
APOPTOSIS	158	−0.31	−1.53	0.000	0.010

GS, signaling pathway name; SIZE, total number of genes under this pathway; ES, enrichment score; NES, normalized enrichment score; NOM *P*-value, represents the confidence of the enrichment result;, and FDR *q*-value, *P*-value corrected for multiple hypothesis testing.

## 4 Discussion

As the most common and malignant histological subtype of kidney cancer, ccRCC has had a continued rise in incidence in recent years. It is widely accepted that a metabolic hallmark of cancer cells is lipidomic remodeling, which broadly encompasses alterations in fatty acid transport de novo lipogenesis, storage as lipid droplets, and β-oxidation to generate ATP [20]. ccRCC is a highly metabolic tumor, which is composed of the appearance of “transparent cells.” The reason for cell forming is the excessive accumulation of a large amount of lipid and glycogen deposits filling the cytoplasm of tumor cells [21,22]. In recent years, research has verified that metabolic adaptation in ccRCC leads to the activation of lipid storage pathways, which is a necessary step in the development of malignant tumors [23]. Therefore, it is necessary to explore more deeply the role of additional molecules in lipid metabolism reprogramming.

ACOX1 is the enzyme required for the first step of peroxisomal β-oxidation, which catalyzes the desaturation of acyl-CoA to 2-trans-enoyl-CoA. It plays a crucial role in lipid metabolism, involved in various aspects of lipid metabolism, including lipid synthesis and catabolism, bile acid synthesis, α-oxidation of branched-chain fatty acids, and β-oxidation of very long fatty acids [24,25]. In humans, ACOX1 deficiency causes impaired peroxisomal β-oxidation, producing a rare neuroinflammatory and neurodegenerative peroxisomal disease, pseudoneonatal adrenoleukodystrophy [26,27]. Some studies have shown that ACOX1 activity increasing due to SIRT5 expression downregulated in primary hepatocellular carcinoma cancer results in poor HCC survival [28]. Abnormal upregulation of ACOX1 by PPAR activation was reported to stimulate hepatic fatty acid oxidation, resulting in excess energy burning in the liver and contributing to the development of liver cancer in rodents [29,30]. In our study, we found that ACOX1 mRNA and protein levels were downregulated in ccRCC through bioinformatic analysis and experimental validation. This leads us to speculate that ACOX1 might act as a tumor suppressor in ccRCC as well.

Because of highly metastasizing, it is necessary to screen out biomarkers with high efficacy for the diagnosis and prognosis of ccRCC and explore the valuable targets for therapy, to improve the life expectancy of patients [31]. To date, a large number of biomarkers have been validated. p53 Overexpression is indicative of a poor prognosis in ccRCC [32]. CD146, one of the cell adhesion molecule family, is overexpressed in ccRCC and associated with poor prognosis [33]. Recently, CTHRC1, NOP2, and P4HB have shown a strong ability to diagnosis and prognosis for ccRCC [34,35,36]. ACOX1 can be combined with MMP1, suppressor of cytokine signaling 3 (SOCS3) to diagnose oral squamous cell carcinoma (OSCC), and downregulation in bladder cancer might be due to PDK1 down-regulation, suggesting that it might serve as a potential biomarker and therapeutic target for bladder [37,38]. In this study, the ROC curves demonstrated that ACOX1 might serve as a diagnostic marker for ccRCC. ACOX1 mRNA expression was correlated with T and M stages and pathological grading by analyzing the TCGA data and the clinicopathological characteristics of patients. OS and DFS illustrate that a lower expression level of ACOX1 is associated with a worse prognosis in patients with ccRCC. In a word, we report that ACOX1 is a potential diagnostic and prognostic molecular biomarker of ccRCC, both at mRNA and at protein levels. However, it is worth further evaluation in larger clinical sample sizes.

Recent studies have shown that acidosis-induced activation of TGF-β2 promotes partial EMT and fatty acid metabolism [39]. Epithelial cells acquired strengthened motility through EMT, which plays a crucial role in initiating ccRCC metastasis [40]. We analyzed the correlation between ACOX1 and the EMT-related genes and found a positive correlation with cadherin 1 (CDH1), CDH2, cyclin-dependent kinase-like 2 (CDK2), and epithelial cell adhesion molecule, and a negative correlation with matrix metalloproteinase MMP9 and vimentin, suggesting that ACOX1 might be regulated by EMT and affect its metastasis in ccRCC.

Epigenetic mechanisms, including DNA methylation, histone modification, and microRNA (miRNA), are currently receiving much attention [41]. miRNA regulates the expression of numerous metabolic genes and can produce heritable phenotypic changes without a shift in DNA sequence. More than 40 miRNAs are associated with urological cancers and several oncogenic pathways, especially in apoptosis, proliferation, EMT, and angiogenic signaling, all of which are regulated by multiple miRNA targets [42]. We observed that miR-16-5p was negatively correlated with ACOX1 expression in ccRCC and that miR-16-5p can target a fixed sequence of the ACOX1 gene, suggesting that it may be one of the mechanisms underlying ACOX1 downregulation in ccRCC. Ding et al. reported that miR-103-3p negatively regulates ACOX1 in NAFLD and suggested a potential therapeutic target [43]. In OSCC miR-31-5p regulates ACOX1 and promotes OSCC migration and invasion by affecting lipid metabolism, thereby triggering intracellular signaling changes [44]. In a study by Li et al., miR-15a could promote adipocyte differentiation by targeting ACOX1, sterol carrier protein 2, and acetyl-coenzyme A acyltransferase 1 in the PPAR signaling pathway [45]. Sun et al. also reported that SIRT in colorectal cancer (CRC) could inhibit miR-15b-5p transcription and then restore ACOX1 expression, through which SIRT1 promotes fatty acid oxidation and inhibits CRC metastasis, suggesting a potential target for metastable CRC therapy [46]. Sequencing studies showing that miRNAs have a central role in the physiological regulation of RCC, with dysregulation, is widespread in RCC [47]. As a conserved endogenous non-coding RNA molecule, miRNA affects various cellular processes, including cell development, differentiation, apoptosis, and proliferation [48]. These findings reveal that miRNAs play a crucial role in regulating tumor development and that miR-16-5p might play a role in ccRCC by regulating ACOX1 expression.

In conclusion, our study demonstrates that ACOX1 is downregulated in ccRCC, revealing its potential significance for the diagnosis and prognosis of ccRCC, and we find that miR-16-5p might act as an upstream regulator to suppress the expression of the ACOX1 gene in ccRCC. In addition, a limitation of this study is the lack of validation of the biological function of ACOX1 in ccRCC, which will provide directions for future studies. Therefore, further efforts are still needed.

**Table 4:** mRNA Expression of hsa-miR-16-5p and its correlation with clinicopathological parameters of patients with ccRCC

		Relevant expression of ACOX1			
Clinicopathological parameters		*n*	Mean ± SD	*t*	*P*-value
Tissue	Normal	70	8.23 ± 0.488	12.075	<0.0001*
	ccRCC	240	9.18 ± 0.61		
Age	<60	114	9.64 ± 0.48	−0.935	0.351
	≥60	146	9.70 ± 0.54		
Gender	Male	97	9.65 ± 0.45	−0.670	0.504
	Female	162	9.69 ± 0.55		
T	T1–T2	156	9.63 ± 0.56	−1.758	0.080
	T3–T4	103	9.74 ± 0.44		
N	No	123	9.67 ± 0.50	0.150	0.881
	Yes	9	9.64 ± 0.37		
M	No	221	9.66 ± 0.55	−0.840	0.402
	Yes	38	9.74 ± 0.27		

SD, standard deviation; ccRCC, clear cell renal clear cell carcinoma; T, tumor; N, lymph node; M, metastasis. *P < 0.05 was considered statistically significant.
